# Investigating the Unexpected Effect of Bulkheads in a Dementia Model of Mice Through Molecular Analysis of the Hippocampus

**DOI:** 10.7759/cureus.72272

**Published:** 2024-10-24

**Authors:** Takuya Sakamoto, Tadashi Ueda, Tetsuhiro Horie, Daisuke Sakamoto, Yasuo Yoshitomi, Yasuhito Ishigaki, Munenori Ono, Nobuo Kato, Tsugiyasu Kanda, Yuji Kasamaki

**Affiliations:** 1 Medical Research Institute, Kanazawa Medical University, Ishikawa, JPN; 2 Department of Pharmacy, Kanazawa Medical University Hospital, Ishikawa, JPN; 3 Department of Community Medicine, Kanazawa Medical University, Ishikawa, JPN; 4 Department of Cardiovascular Surgery, Kanazawa Medical University Hospital, Ishikawa, JPN; 5 Department of Biochemistry, Kanazawa Medical University, Ishikawa, JPN; 6 Department of Physiology, Kanazawa Medical University, Kahoku, Ishikawa, JPN; 7 Department of Physiology, Kanazawa Medical University, Ishikawa, JPN; 8 Department of General Medicine, Kanazawa Medical University Himi Municipal Hospital, Toyama, JPN

**Keywords:** alzheimer’s disease, apoe, covid-19, dementia, hippocampus

## Abstract

Aim: This study aimed to evaluate the impact of long-term exposure to physical barriers used as preventive measures during the coronavirus disease 2019 (COVID-19) pandemic on cognitive function and behavior in an apolipoprotein E^−/−^ (ApoE^−/−^) mouse dementia model.

Methods: ApoE^−/−^ mice were divided into co-housed, partitioned by a transparent bulkhead (partitioned), and isolated groups. To assess anxiety, cognitive recognition, and spatial learning, behavioral tests, including the open-field test, novel object recognition test, and Morris water maze test, were conducted at three and six months after the start of the 33-week rearing period. RNA-sequencing analysis of hippocampal tissues was performed to investigate gene expression changes.

Results: The partitioned group exhibited reduced exploratory behavior, lower recognition index, and impaired spatial learning compared with the co-housed group. However, the differences were not significant. Based on morphological analysis, the partitioned and isolated groups presented a significant reduction in neuronal density in the hippocampal CA1 region. RNA-sequencing analysis showed significant changes in the expression of genes related to neurotransmitter transport, neurite outgrowth, and neuropeptide signaling pathways.

Conclusions: Prolonged physical isolation, even with visual contact, can adversely affect cognitive function and hippocampal structure in dementia models. Changes in gene expression indicate that neurotransmitter imbalances and neuroinflammatory responses may contribute to these effects. These findings emphasize the need to develop new infection-prevention measures for patients with dementia during the COVID-19 pandemic.

## Introduction

In Japan, single-person households account for 34% of all households, and living alone in a single-person household contributes to an increased risk of social isolation. A 2016 survey conducted by the Cabinet Office revealed that 7.0% of elderly individuals living alone in Japan reported having “very little” conversation with others, a figure significantly higher than that in other countries [1]. In 2022, individuals aged ≥65 years accounted for 29% of the overall population. Globally, by 2050, the number of people aged ≥65 years is projected to double that of children aged ≤5 years [2]. Social isolation is associated with various adverse health outcomes, including neuronal loss, impaired neuronal repair, increased inflammation, anxiety and depression, sleep disturbances, and impaired vascular regulation.

During the coronavirus disease 2019 (COVID-19) pandemic, which was caused by severe acute respiratory syndrome coronavirus 2, various preventive measures were implemented to inhibit the spread of the virus [3]. Among these measures, the use of personal protective equipment and physical barriers, such as bulkheads, became common for reducing direct human contact and potential transmission during activities such as meetings and meals [4-6]. Due to the COVID-19 pandemic, contact among humans has been severely restricted. In elderly people and patients with underlying medical conditions who are at high risk of dementia, rapid deterioration in cognitive function has been a cause of concern. The abovementioned interventions have been effective in reducing the spread of infection; however, their long-term impacts on cognitive function and behavior remain unclear.

Dementia, particularly Alzheimer’s disease, is characterized by progressive cognitive decline and is associated with neuropathological features, such as amyloid-β plaque formation, neuroinflammation, and synaptic loss [7]. Apolipoprotein E^−/−^ (ApoE^−/−^) mice, which lack the gene encoding ApoE, are a well-established model for assessing Alzheimer’s disease due to their susceptibility to these neuropathological features [8].

Previous studies have shown that social isolation and reduced environmental stimulation can exacerbate cognitive decline in dementia models [9, 10]. However, the specific effects of physical barriers, such as those used during the COVID-19 pandemic, on cognitive function and behavior in dementia models have not been comprehensively investigated.

The present study aimed to evaluate the impact of COVID-19 infection prevention measures, including the use of acrylic partitions, on cognitive function via histopathological analysis, genetic analysis, and physiological function tests.

## Materials and methods

Experimental design

The mice used in our study were an APOE-deficient model (APOE^-/-^) known as the KOR/StmSlc-Apoe^shl^ mice, generated by introducing the hyperlipidemia-associated Apoe^shl^ gene into the genetic background of BALB/c mice. This model was developed at the Clinical Oncology Research Institute of the Saitama Prefectural Cancer Center, using mice originally derived from Koriyama, Japan, and is widely used in various studies as a model for dementia.

Twelve six-week-old male C. KOR/Stm Slc-Apoe^shl^ (ApoE^−/−^) mice (Japan SLC, Inc., Shizuoka, Japan) were housed under controlled conditions (22°C, 12-h light/dark cycle) with ad libitum access to water and chow. Mice were grouped as co-housed (two per cage), partitioned (two per cage with acrylic separation), isolated (one per cage), and maintained for 33 weeks. Behavioral tests were conducted at three and six months, and hippocampi were collected post-experiment. All animal experiments were performed in accordance with the Institutional Guidelines of Kanazawa Medical University (KMU) and the guiding principles of the Physiological Society of Japan. These experiments were approved by the Institutional Animal Care (approval number: 2023-43) and Use Committee of KMU and were performed at the Animal Care Center of KMU.

Histological analysis

All mice were euthanized with isoflurane and decapitated. Brain weights were measured [11], and hippocampi were excised [12], fixed in 10% formalin, and processed for histological analysis. Hematoxylin and eosin (H&E) staining was performed, and hippocampal regions were assessed via phase-contrast microscopy. ImageJ software was used to calculate cell density (cells/mm²) by measuring the area and counting cells at three randomly selected points.

Behavioral tests

Open-Field Test (OFT)

The OFT is an experimental method used to assess general locomotor activity, anxiety, and exploratory behavior in rodents. In this experiment, mice were placed in a circular open field (80 cm in diameter, 50 cm in height), which was divided into two zones: an outer zone (zone 1) and an inner zone (zone 2). Zone 2 was defined as a circular area with a diameter of 55 cm located at the center of the field, while the remaining area was referred to as Zone 1. The mice were allowed to move freely within the field for 10 minutes, and their movements were recorded using the SMART v 2.0 system (Panlab/Harvard Apparatus, Holliston, MA, USA). The percentage of time spent in the inner zone (time in center = inner zone time / total time × 100%) was then analyzed.

Novel Object Recognition Test

The novel object recognition test is based on the spontaneous exploration behavior of mice, particularly their tendency to explore novel objects over familiar ones. Each mouse was provided with a three-minute habituation period in an empty box (40 × 25 × 20 cm), followed by a five-minute rest. After the resting period, a three-minute training session was conducted, during which the mice explored two identical objects (object A and object A1) placed 10 cm away from the walls in two adjacent corners. Exploration behavior was defined as touching or sniffing objects with the nose or forepaws. The exploration time for each object was recorded. Twenty-four hours after the training session, the less interesting object was replaced with a new object (object B), and all mice were allowed to explore the box for three minutes with the familiar object (object A or A1) and the new object (object B). The recognition index was calculated as TB / (TA + TB), where TA is the exploration time for the familiar object A and TB is the exploration time for the new object B.

Morris Water Maze Test for Spatial Memory Assessment

The Morris water maze test was used to evaluate spatial memory. Maze training was conducted between 9:00 AM and 12:00 PM, utilizing a circular pool with a transparent Plexiglas platform submerged 10 mm below the water surface. All mice participated in forced swimming sessions four times a day for five consecutive days. The pool was divided into four quadrants. In each session, the mice were placed in one of the four quadrants at the edge of the pool. The session ended when the mouse reached the platform and remained there for five seconds or after a maximum of 60 seconds had elapsed, whichever came first. A 30-second rest period was provided between each session. After the final experiment, the platform was removed, and a 60-second trial was conducted. The area where the platform was located was designated as quadrant 2 and the remaining area as quadrant 1. The time spent in quadrants 1 and 2 was evaluated within a 60-second period. The swimming paths of the mice were analyzed using SMART v 2.0, and the proportion of time spent in the target quadrant was evaluated.

RNA-sequencing (RNA-seq) analysis

Hippocampal tissues were harvested from mice, followed by total RNA (RiboNucleic Acid) extraction, cDNA (complementary DNA) synthesis, and RNA sequencing (RNA-seq), which were performed at Bioengineering Lab. Co., Ltd. (Sagamihara, Kanagawa, Japan). Briefly, cDNA libraries were constructed using the MGIEasy RNA Directional Library Prep Set (MGI Tech Co., Ltd., China), following the manufacturer’s protocol. The fragments were sequenced on the DNBSEQ-T7 sequencer (MGI Tech Co., Ltd.). Data analysis that included adapter trimming with Cutadapt (developed by Marcel Martin, NBIS: National Bioinformatics Infrastructure Sweden, Uppsala, Sweden), quality filtering with sickle, read mapping to the mouse reference genome with STAR (Spliced Transcripts Alignment to a Reference, version 2.7.9a, developed by Alexander Dobin, Cold Spring Harbor Laboratory, New York, USA), format conversion and sorting with Samtools, read counting and normalization with feature counts and TPM (Transcripts Per Million), differential expression analysis using TCC (Tag Count Comparison, version 1.34.0, developed by Jianqiang Sun, University of Tokyo, Tokyo, Japan) and edgeR (empirical analysis of digital gene expression data in R, version 3.36.0, developed by Yunshun Chen, WEHI, Parkville, Australia), and visualization with a volcano plot generated via bioinfokit of Python. GO analysis (Gene Ontology analysis) was conducted using the DAVID (Database for Annotation, Visualization and Integrated Discovery) website (https://david.ncifcrf.gov/). 

Statistical analysis

Statistical analysis was performed using GraphPad Prism (version 9.4.1; GraphPad Software Inc., San Francisco, CA, USA). The results are expressed as mean ± standard deviation. Comparative analyses of the behavior test results and body and brain weights were performed using a one-way analysis of variance, followed by Tukey’s multiple comparison test. A p-value of <0.05 was considered statistically significant. As shown in the Go analysis, p-values were calculated using Fisher’s exact test. The term “#Molecules” represents the molecules that fit into the GO term.

## Results

Longitudinal changes in the body weight of the mice

**Figure 1:**
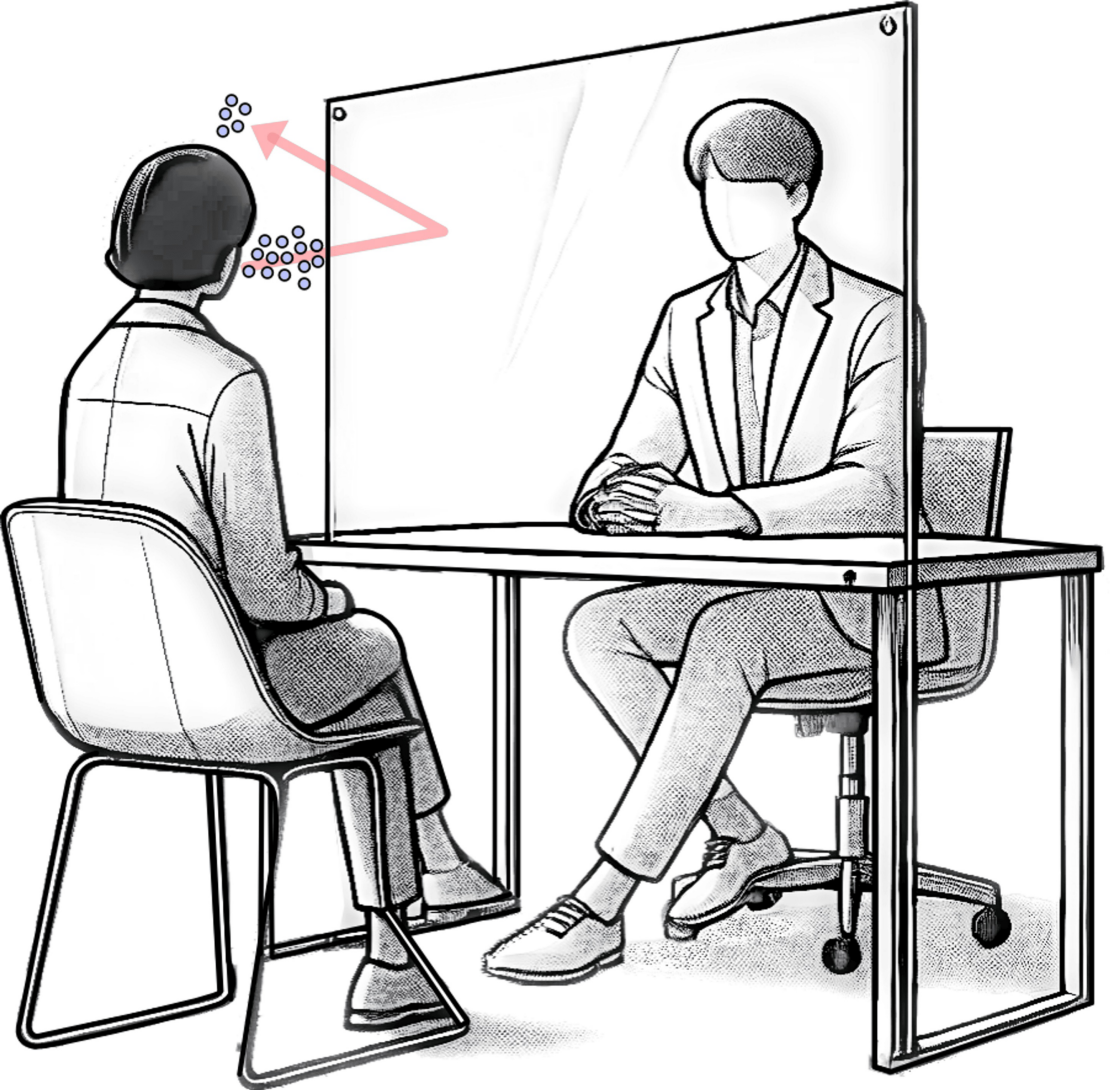
Infection control measures during the COVID-19 pandemic in Japan. In Japan, transparent acrylic partitions were widely used as infection prevention measures during the COVID-19 pandemic, and communication between individuals was always conducted through these acrylic partitions. This illustration was created by the author using Affinity Designer (Serif Europe Ltd., Nottingham, United Kingdom).

Bulkheads, commonly known in Japan as transparent acrylic partitions, were used as infection prevention measures during the COVID-19 pandemic. They were widely installed in public spaces such as offices, restaurants, and schools to reduce the risk of droplet transmission by creating a physical barrier while still allowing visual communication (Figure 1).

**Figure 2:**
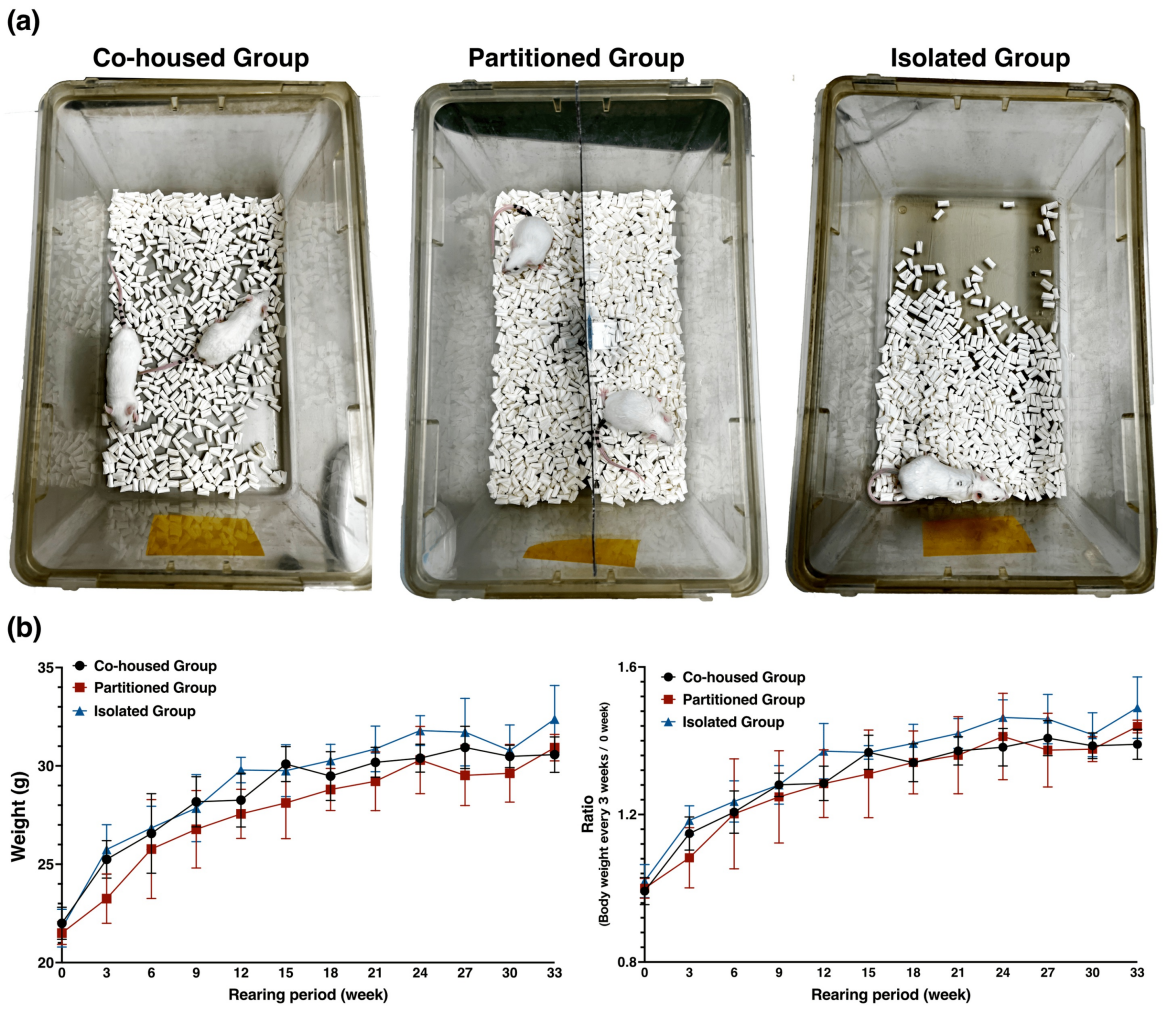
Longitudinal changes in the body weight of mice (a) Experimental housing conditions for apolipoprotein E−/− mice. (b) Six-week-old apolipoprotein E−/− mice, each reared under different housing conditions, were monitored over a 33-week period. Their body weight changes were assessed at three-week intervals (n = 4). The left graph shows the changes in body weight, and the right graph shows the ratio compared to the start of the rearing period (0 weeks). Values are presented as mean ± standard deviation.  At the end of the 33-week rearing period, significance testing using a one-way analysis of variance, followed by Tukey’s multiple comparison test was conducted, and no significant differences were found between the groups. The F-values from the one-way ANOVA were 2.579 on the left graph and 3.315 on the right graph.

The study employed three distinct groups (Figure 2a), namely, co-housed, partitioned, and isolated, to model various social conditions that may occur in human societies, particularly under circumstances such as social isolation experienced during the COVID-19 pandemic.

The longitudinal changes in the body weight of mice were measured during the 33-week rearing period. There were no significant differences in body weight changes among the co-housed, partitioned, and isolated groups. However, the isolated group was more likely to have a greater weight gain than the co-housed and partitioned groups (Figure 2b).

Specific behavioral test

**Figure 3:**
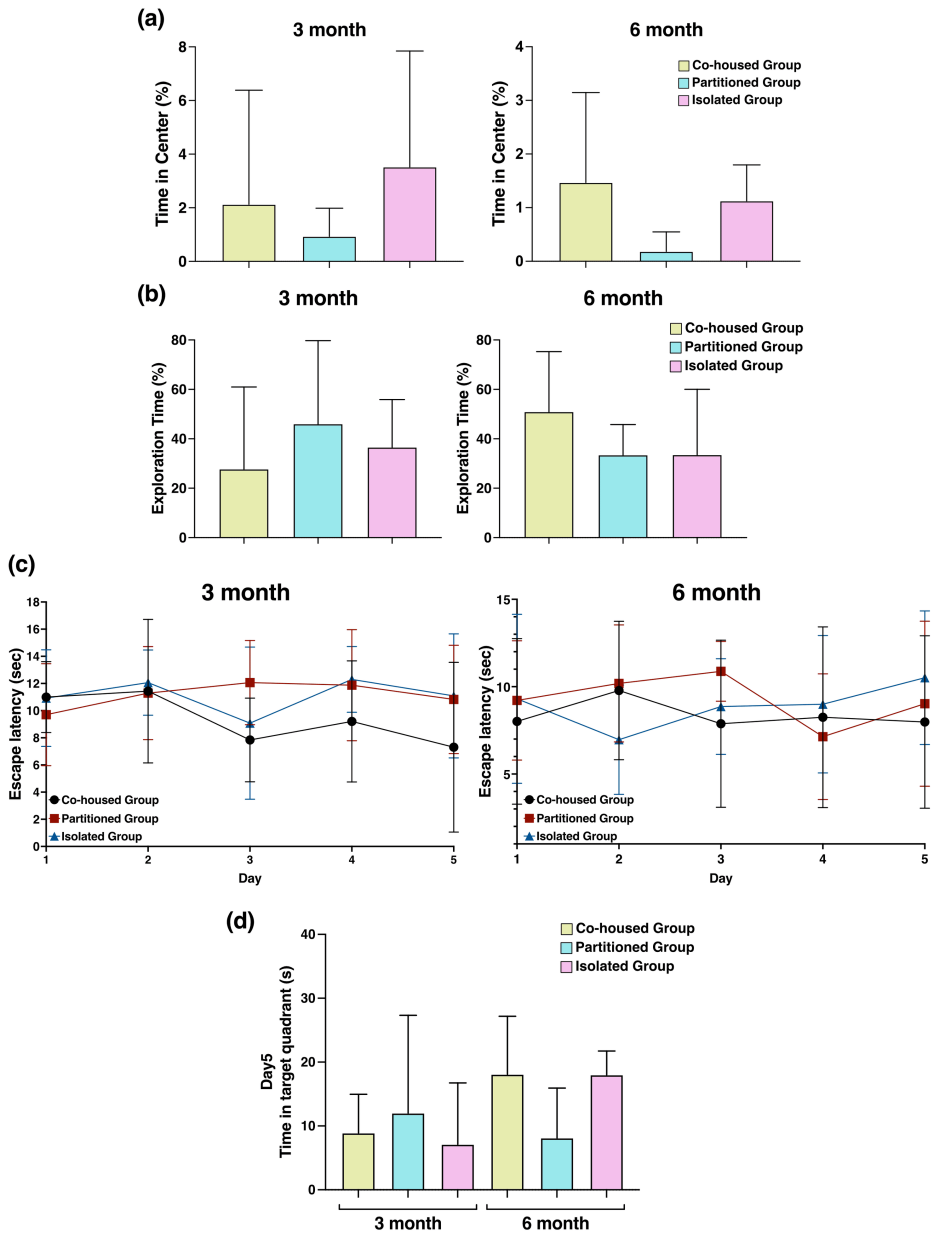
Behavioral test using the open-field test, novel object recognition test, and Morris water maze test (a) The open-field test shows the percentage of time spent in the center of an open field in all housing conditions (n = 4). The partition group exhibited a reduced center time compared with the co-housed and partitioned groups. (b) The novel object recognition test was conducted to evaluate cognitive function, without significant differences in the recognition index among the housing conditions (n = 4). The Morris water maze test was conducted to examine the spatial learning and memory abilities of the mice. This test was performed in a circular pool with an underwater platform, and the mice underwent four forced swimming sessions per day for 5 consecutive days. (c) The time taken to reach the platform (escape latency) and (d) the time spent in the target quadrant (quadrant 2) on day 5 were recorded and analyzed (n = 4).  Data are presented as mean ± standard deviation. Statistical significance was assessed using one-way analysis of variance, followed by Tukey’s multiple comparison test. There were no significant differences among the co-housed, partitioned, and isolated groups. The F-values from the one-way ANOVA were 0.531 in the left graph and 1.564 in the right graph in Figure 3a. Similarly, in Figure 3b, the F-values were 0.384 in the left graph and 0.841 in the right graph. The F-value in Figure 3d was 1.12.

The OFT is one of the most commonly used tests to measure exploratory behaviors and general activity levels [13]. The mice in each housing condition underwent the OFT at three and six months of age. The partitioned group was more likely to have a reduced percentage of time spent in the central area compared with the co-housed group at the six-month time point. However, the difference was not statistically significant (Figure 3a). The novel object recognition test assessed the cognitive function of mice in different housing conditions by comparing the exploration time of a novel object and that of a familiar object. The recognition index, calculated as TB / (TA + TB), did not significantly differ among the co-housed, partitioned, and isolated groups (Figure 3b).

To evaluate the spatial memory and learning abilities of the mice, the Morris water maze test was conducted. This test utilized a circular pool with a hidden platform submerged below the water surface. The mice underwent training sessions four times a day for five consecutive days. The primary recorded metrics were the escape latency (time taken to find the platform) (Figure 3c) and the time spent in the target quadrant 2, where the platform was previously located, during the probe trial conducted on the fifth day (Figure 3d). There were no significant differences in terms of the primary recorded metrics under any of the housing conditions.

Morphological changes in the mouse brain

**Figure 4:**
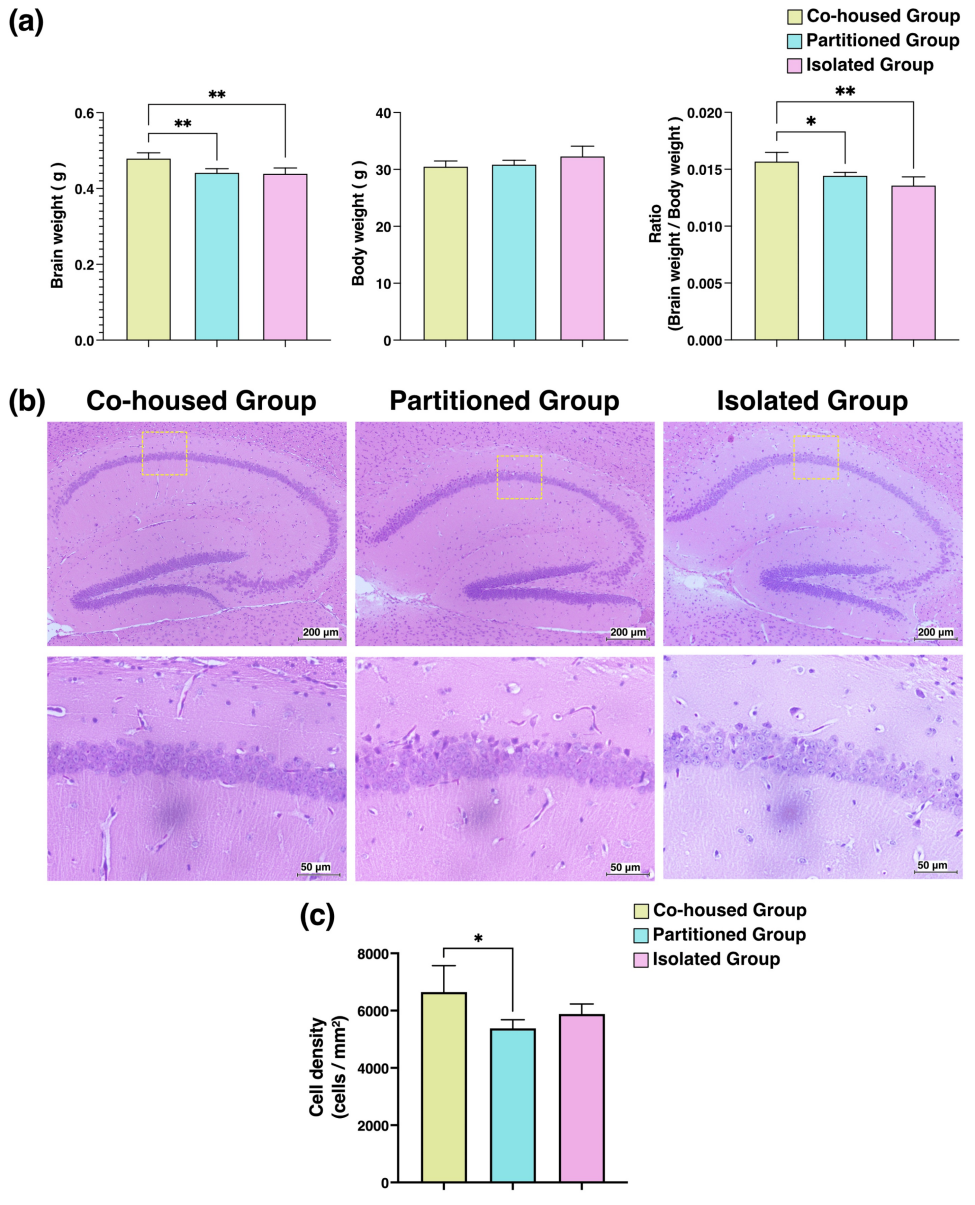
Histological changes in the hippocampus of mice based on hematoxylin and eosin (H&E) staining (a) The body weights of the mice were measured at the end of the 33-week rearing period, and the brains of the mice from each group were harvested and weighed. (b) H&E-stained images of the hippocampus of the brains of mice under each housing condition. The enlarged images of the areas outlined by yellow dotted lines are shown in the lower panels. (c) The cell density of the hippocampal region of mice in each housing condition. In the CA1 region, which plays an important role in the formation of spatial and episodic memory, three random points on H&E-stained images were captured using phase-contrast microscopy. The area and number of cells in densely populated regions were measured to assess cell density. *p < 0.05, ** p < 0.01 vs. the co-housed group.

After the 33-week rearing period, the body weight of mice from each group was measured. Next, the mouse brains were extracted and weighed. The brain-to-body weight ratio was analyzed to investigate the effects of different housing conditions on brain weight. The brain-to-body weight ratio of the partitioned and isolated groups significantly decreased compared with that of the co-housed group (Figure 4a).

Histological examinations of the hippocampal regions of the brains of the mice from each experimental group were performed. H&E staining was conducted to evaluate morphological changes in and the cell density of the hippocampus, with a particular focus on the CA1 (Cornu Ammonis 1) region, which is important for memory formation and spatial navigation. The partitioned group presented with a fewer number of neuronal cells and exhibited signs of neuronal atrophy compared with the co-housed group (Figure 4b). Quantitative analysis revealed that the cell density of the hippocampal CA1 region of the partitioned group significantly reduced compared with that of the co-housed group (Figure 4c).

Comprehensive gene expression analysis using RNA-seq

**Figure 5:**
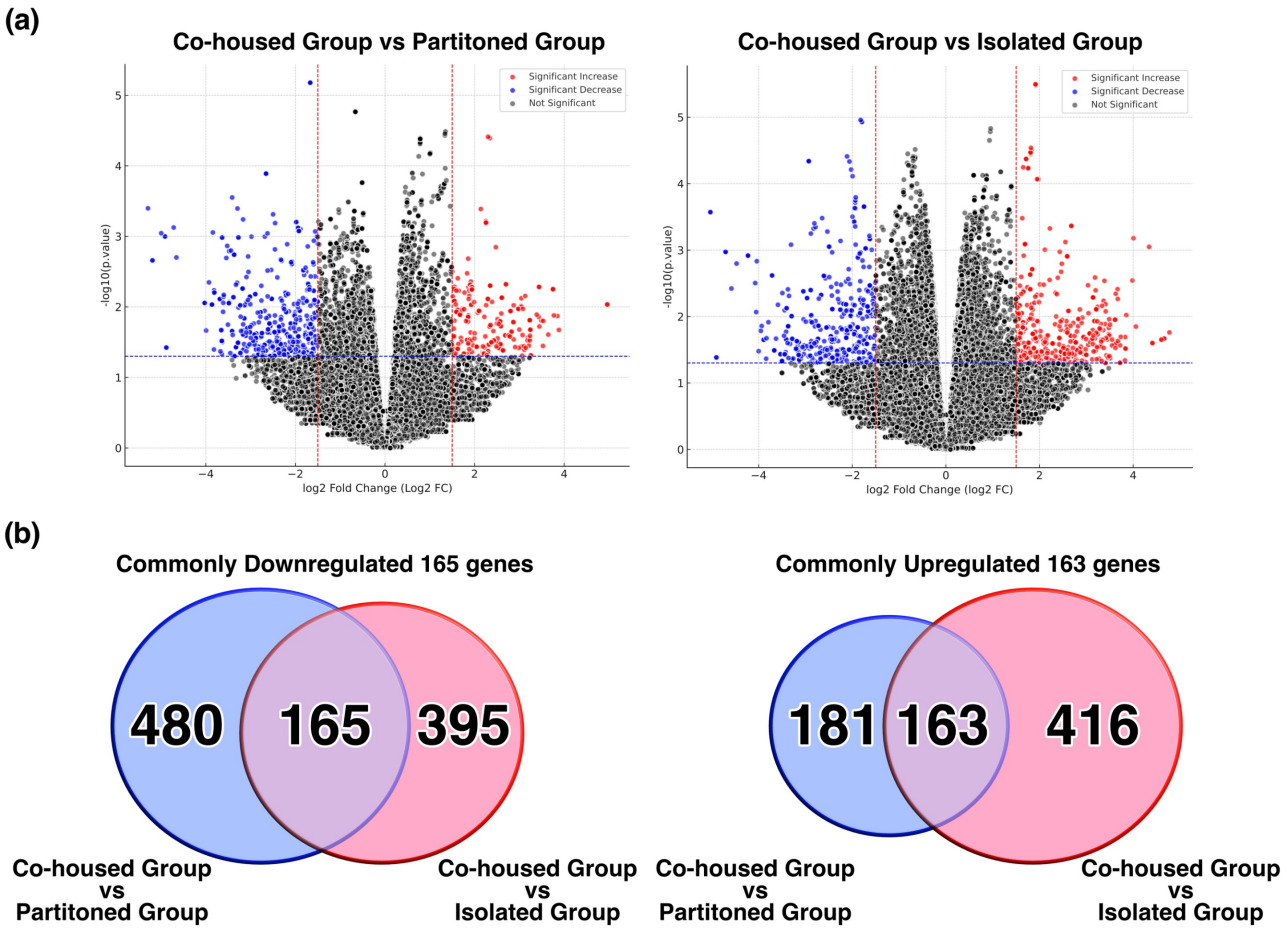
Comprehensive gene expression analysis of the hippocampus of mice The hippocampi were extracted from the mice in the co-housed, partitioned, and isolated groups. The total RNA was extracted, and RNA-sequencing analysis was performed (n = 4). (a) Volcano plots were created to illustrate the gene expression changes in each housing condition compared with the co-housed group. Genes with a significantly altered expression between the two groups were presented as dotted lines in the graph based on specific criteria (p-value < 0.05 and log2 fold change > |1.5|). (b) Venn diagrams were used to show the genes that were commonly expressed between the co-housed and partitioned groups and between the co-housed and isolated groups.

The partitioned group exhibited brain atrophy, unlike the co-housed group. To investigate the genetic factors associated with brain atrophy, the hippocampus of the brain was harvested, and the total RNA (ribonucleic acid) was extracted for RNA-seq analysis (n = 4). Genes that showed significant expression changes between the co-housed and partitioned groups and between the co-housed and isolated groups were extracted using specific criteria (p-value < 0.05 and log₂ fold change > |1.5|) (Figure 5a). The comparison between the co-housed and partitioned groups revealed 645 genes with significantly decreased expression and 344 genes with increased expression. The comparison between the co-housed and isolated groups revealed 560 genes with significantly decreased expression and 579 genes with increased expression. In both comparisons, 165 genes were commonly downregulated, and 163 genes were commonly upregulated (Figure 5b).

Next, we focused on the genes that commonly changed in both comparisons and performed GO analysis using DAVID to provide an overview of the expression changes. Table 1 shows the main gene categories with altered expression. The analysis identified 165 commonly downregulated genes involved in neural functions, while the upregulated genes included those associated with sequence-specific double-stranded DNA (deoxyribonucleic acid) binding, multicellular organism development, regulation of DNA-templated transcription, and protein kinase binding.

**Table 1:** Gene Ontology analysis GO analysis (Gene Ontology analysis) was performed on the genes that showed common changes in both comparisons of expression shown in Figure 5b. The table presents the main categories of genes that exhibited significant changes. The p-values were calculated using Fisher’s exact test. The p-value was calculated using Fisher’s exact test.

Gene Ontology analysis of the extracted downregulated genes (165 genes)				
ID	Description	−log10 (p-value)		#Molecules
GO:0005326	Neurotransmitter transmembrane transporter activity	3.19	SLC17A8, SLC6A2, SLC6A4	
GO:0051899	Membrane depolarization	4.07	CHRNA2, CHRNA5, PHOX2B, SLC6A4	
GO:0043005	Neuron projection	4.61	TPH2, TPH1, CHRNA2, CHRNA5, NPY4R, SLC17A8, SLC6A2, SLC6A4	
GO:0007218	Neuropeptide signaling pathway	5.75	POMC, NPPB, NPY4R, AGRP, PTH2, QRFP	
Gene Ontology analysis of the extracted upregulated genes (163 genes)				
ID	Description	−log10 (p-value)	#Molecules	
GO:1990837	Sequence-specific double-stranded DNA binding	3.28	GCM2, GBX2, CDX1, SHOX2, PITX1, RUNX3	
GO:0007275	Multicellular organism development	2.71	GCM2, EDARADD, CDX1, SHOX2, WNT9B, EPHA1, PITX1	
GO:0006355	Regulation of DNA-templated transcription	2.31	GCM2, TCF7L2, GBX2, CDX1, SHOX2, PITX1, RUNX3	
GO:0019901	Protein kinase binding	2.25	CHRNA2, TCF7L2, CD4, PRKCD, EPHA1, TTN	

## Discussion

This study used a dementia mouse model to evaluate the impact of physical barriers used as preventive measures during the COVID-19 pandemic on cognitive function and behavior. The ApoE^−/−^ mouse model is a prominent dementia model characterized by significant amyloid-β plaque formation, neuroinflammation, and vascular dysfunction [8]. In addition, the hippocampal neurons of ApoE^−/−^ mice are susceptible to synaptic loss as a result of cytotoxicity being associated with aging [14-16]. We hypothesized that the use of bulkheads, even with visual contact, can negatively affect cognitive function and behavior by reducing physical and social interaction. To test this hypothesis, the ApoE^−/−^ mice were divided into the co-housed, partitioned, and isolated groups.

To assess anxiety, cognitive recognition, and spatial learning, behavioral tests, including OFT, novel object recognition test, and Morris water maze test, were conducted at three and six months after the initiation of the 33-week rearing period (Figure 3). Although the differences were not statistically significant, the partitioned group exhibited reduced exploratory behavior, lower recognition index, and impaired spatial learning compared with the co-housed group. Fuentes et al. comprehensively analyzed age-related behavioral changes in ApoE^−/−^ mice and assessed the role of ApoE in cognitive function and motor coordination. Notably, ApoE^−/−^ mice aged ≥12 months (48 weeks) showed a decline in cognitive functions, such as exploratory behavior and memory retention [17]. In the present study, ApoE^−/−^mice aged up to 39 weeks were used, which may explain the lack of significant differences observed at this relatively early stage. However, in all evaluations, the partitioned group showed cognitive decline compared with the co-housed and isolated groups. Thus, living in a barrier-separated environment may accelerate the progression of cognitive impairment.

The hippocampus, located in the temporal lobe of the brain, plays an essential role in memory formation and spatial recognition, and it is a focal point in the study of neurodegenerative diseases such as Alzheimer’s disease [18,19]. The hippocampus is divided into several regions, with the CA1 (Cornu Ammonis 1) region being particularly significant for information integration and transmission, synaptic plasticity, and pathological studies of neurodegenerative diseases. Furthermore, the CA1 region is significantly susceptible to the early-stage effects of Alzheimer’s disease [20-22]. As shown in Figure 4, unlike the co-housed group, the partitioned and isolated groups exhibited morphological changes in the hippocampus, with a significantly reduced neuronal density in the CA1 region. Based on these results, physical separation, even with visual contact, may negatively affect the hippocampal structure.

Based on a comprehensive gene expression analysis using RNA-seq, the partitioned and isolated groups exhibited significant downregulation in the expression of genes related to neurotransmitter transport, neurite outgrowth, and neuropeptide signaling pathways compared to the co-housed group (Figure 5, Table 1). Among the commonly downregulated genes, the solute carrier (SLC) superfamily, including *SLC17A8 (solute carrier family 17 member 8)*,* SLC6A2 (Solute Carrier Family 6 Member 2)* and *SLC6A4 (Solute Carrier Family 6 Member 4)​​​*​​​​, plays an important role in the reuptake and synaptic concentration regulation of neurotransmitters such as GABA (Gamma-Amino Butyric Acid), glutamate, serotonin, dopamine, and norepinephrine. These transporters are involved in neurodegenerative diseases such as Alzheimer’s disease and depression [23]. Therefore, the partitioned group, similar to the isolated group, may present with neurotransmitter imbalances due to SLC transporter abnormalities, thereby affecting cognitive function.

Among the commonly upregulated genes (Table 1), *GCM2* stands out as an anti-inflammatory transcription factor expressed in specific microglia. Pavilidaki et al. showed that the expression of *GCM2* increases in response to chronic inflammation associated with aging, playing a protective role against the development and progression of neurodegenerative diseases caused by suppressing neuroinflammation [24]. Therefore, some commonly upregulated genes may have protective mechanisms against the progression of cognitive decline.

Contact with others improves loneliness in elderly individuals [25]. Cognitive impairment caused by social isolation has also been observed in mouse models [26], and social contact with a partner has a positive effect. These results highlight the importance of environmental and social factors in dementia progression during the COVID-19 pandemic. Barriers are beneficial for infection prevention; however, they may hinder social interactions and contribute to cognitive decline. Hence, their impact on mental health should be considered. This study emphasizes the need to establish interventions balanced between physical and mental health for patients with dementia.

This study has several limitations that should be acknowledged. The sample size of mice was limited, and the experimental period was restricted to 33 weeks, which may not fully capture the long-term effects of physical separation on cognitive function. Longer observational periods are required to evaluate the chronic impact of social isolation or environmental barriers on dementia progression. In addition, while the bulkhead model used in this study was designed to mimic physical isolation, it does not fully replicate the complexity of social isolation experienced by humans, especially during the COVID-19 pandemic. Future research should explore more refined experimental models that better simulate human social interactions and environmental factors. 

## Conclusions

This study provides significant insights into the risks associated with prolonged physical isolation during the COVID-19 pandemic on dementia progression. Furthermore, it supports the need to develop new infection prevention methods for patients with dementia. Nevertheless, future research should be conducted to explore the underlying mechanisms of these environmental effects and develop strategies to mitigate their negative impacts.
